# Vaccination in the Light of the Royal British Commission

**Published:** 1897-09

**Authors:** M. R. Leverson

**Affiliations:** Fort Hamilton, L. I., N. Y.


### VACCINATION IN THE LIGHT OF THE ROYAL BRITISH COMMISSION.

M. R. LEVERSON, M. D., FORT HAMILTON, L. I., N. Y.

(Continued from page 301.)

From the number of communications I have received from physicians, I am encouraged to believe that the abstract of the testimony given before the Royal (British) Commission on vaccination, now in the course of publication in THE HOMŒOPATHIC PHYSICIAN, is exciting some amount of interest in the profession. Naturally, the opinions expressed in these letters vary greatly, but all the writers seem to appreciate the effort to place them in possession of the light thrown upon the subject of vaccination by that memorable inquiry.

I have profited by criticisms expressed by several writers that the very words of the witness should be given in important matters, as the readers of THE HOMŒOPATHIC PHYSICIAN can observe by comparing the articles which were published in the numbers for December, 1896, and January, 1897, with such as have appeared later. Among the letters received was one from which I make the following extracts.

It is from a "regular," who looks upon Homœopathy as

"mind cure," but wanted to get at the substance of the testimony before the Commission, and, learning of the abstracts publishing in THE HOMŒOPATHIC PHYSICIAN, has been reading them. After thus introducing himself, he goes on:

"In THE HOMŒOPATHIC PHYSICIAN for December, 1896, you say you 'cannot pretend to be now without an opinion.' May you not now be so prejudiced that, unintentionally of course, your abstract does not fairly represent the witnesses?

"Your abstract of Dr. Thorne's testimony reads more like a very hostile review than a fair abstract, and although you refer by their numbers to the questions and answers of the witness, you know well that verification on the part of nearly all your readers is almost impossible, for the same reasons that render a *fair* abstract desirable. I cannot accept your judgment of Dr. R. Thorne Thorne 'as a noteworthy example of official arrogance, ignorance, and dishonesty,' without further evidence. The gentleman stands too high, at least among the officials and leaders of the profession, to be so dismissed, and I suggest to you the propriety of quoting his own words instead of giving what you claim to be the substance of his statements. I refer particularly to your statements toward the end of page 7 of the January number of THE HOMŒOPATHIC PHYSICIAN."

In accordance with my correspondent's suggestion, I now present an abstract of Dr. Thorne's* testimony, giving his own words in many cases where I did not before do so. It will be for the readers of THE HOMŒOPATHIC PHYSICIAN to judge whether my criticism of Dr. R. Thorne Thorne in the January issue of the journal was too severe.

At Q. 764 Dr. Thorne adopts the report of the Epidemiological Society as to the alleged immunity from small-pox of

---

* I call this gentleman *Doctor* Thorne in accordance with our American practice. He is actually M. B. and a Fellow of the Royal College of Physicians. In this country he would, under similar graduation, be *Doctor* of Medicine.

revaccinated nurses in the small-pox hospitals, and says (p. 38b, First Report of the Royal British Commission of 1889), speaking of the staffs of the Metropolitan Asylum's small-pox hospitals: "Then there remain 655. Now, 645 out of the 655 had not only been vaccinated in infancy, but had also been revaccinated before entering on their duties at the hospitals. *Not a single one of them took the small-pox.*"

[But Dr. C. T. Pearce, testifying before the Parliamentary Committee of 1871, stated: "I yesterday visited the small-pox hospital at Highgate, and was not a little astonished when the door was opened by a nurse whose face was scarified by small-pox. * * * I went from Highgate to Northumberland Street, and there had an interview with the assistant clerk, who gave me the astounding information that at Stockwell a nurse recently engaged because she was pitted with small-pox, was revaccinated on her engagement, and she is now in bed with confluent small-pox."

At Fulham Hospital, three of the revaccinated attendants under Dr. Makuna took small-pox. Dr. Sweeting, one of the reputed "authorities" for the report of the Epidemiological Society, says that four of his revaccinated nurses had taken the disease.

At the Lewis Fever Hospital a nurse was engaged. She had been vaccinated in infancy, revaccinated when ten years of age, and re-revaccinated in November, 1881, on her engagement as nurse. She took small-pox shortly after. The House Surgeon of Fulham Hospital stated in January, 1882, that five of his revaccinated nurses had taken small-pox.

Sister Clara (Miss Lumly), a revaccinated nurse at Guy's Hospital, died of small-pox March 19th, 1892. Four professional nurses in the Perth Infirmary, at least twice vaccinated, took small-pox in November, 1889.

Two revaccinated nurses in the Ashton-Under-Lyne Hospital took small-pox. In the Metropolitan Asylum's Board's annual report for 1894, it is said (page 204): "One male attendant there" (at the Eastern Station) "and one at the Western Station, both of whom had been unsuccessfully (?) revaccinated when appointed, contracted small-pox."

At Q. 801, Dr. Thorne says: "I am afraid that my opinion upon the remote origin of vaccine lymph would be of very little interest to the Commission. I have never studied the question. * * * It has been a matter of little importance to me where it came from, so long as the results as regards small-pox were what they are."

With regard to the source of the supply of calf lymph, with which to originate calf-lymph inoculation, asked if it came within his department (Q. 803), he says: "It did, but I was at that time a traveling inspector in the country, and did not even know of it till I read it in the reports." And (Q. 804) says that Dr. Buchanan will be able to give that information. (He never does.)

Q. 806. "With regard to Dr. Seaton's report to the Local Government Board in 1874, which you put in as evidence, may I take it that in addition to having read the work, you are satisfied of the facts and figures therein contained as being tolerably accurate?" "I accept Dr. Seaton's facts and fiures. *I have not verified them.*"

Q. 807. "On page 9 it is stated: 'The estimated annual small-pox death-rate of England in the last century was 3,000 per 1,000,000 of population.' Can you give us any notion how that was ascertained?" "I cannot. I have not the data on which this report was compiled."

Q. 808. "We have been told that there is no means of ascertaining, with any degree of precision, the population of this country in the last century. If that were the case, I presume the figures of 3,000 per 1,000,000 of the population, could not be vouched with any accuracy?" "It is given here merely as an estimate; I suppose it was estimated from the best means he had."

Q. 809. "You are not aware of the basis on which it was founded?" "I am sorry to say we have not the data upon which Dr. Seaton compiled his work."

Q. 810. "There are several tables given here relating to small-pox in Scotland and Ireland. Could you tell the Commission whether before the epidemic of 1871 Scotland and Ireland were considered to be well-vaccinated countries?" "I could not tell you. I really, as I said at the time, handed this book in and gave one or two of its essential features simply from a historical point of view, as showing the basis for the action which the L. G. B. were taking with regard to vaccination. I have no doubt that at the time the facts and figures were verified, and the Local Government Board have practically accepted them as the trustworthy and honest production of Dr. Seaton."

Q. 811. "In the Blue Book for 1871, at page 253, Q. 4,389, Dr. Wood, in reply to the question, 'Can you state whether the operation of the Act of 1863 has largely diminished the amount of epidemic small-pox in Scotland?' Stated 'very largely,' and gave figures showing the decline from 1,646 deaths from small-pox in 1863, to 15 in 1868; 100 in 1869, and 150 in 1870. Now I find that in the year 1871 there were 1,442 deaths from small-pox in Scotland; in 1872, 2,446; in 1873, 1,126; in 1874, 1,246, making a total in four years of 6,260. Do you think if the decline then claimed for the administration of the vaccination Act of 1863 were attributed to the operation of that Act, that the excessive mortality in the following years would render that previous conclusion an unfair or an unjustifiable one?" "I should imagine that the increase during that period of epidemic was precisely on the same footing as the increase which we had in England, and that the arguments which have been used with reference to England would apply in the same way to Scotland."

Q. 812. "You do not think that there was a tendency there, which possibly has been imitated elsewhere, to claim that when small-pox has subsided coincidentally with the extension of vaccination, vaccination has been responsible for stamping it out, whereas, a subsequent epidemic has disproved that observation?" "I should say that no epidemic that we have any actual details about, has ever disproved the value of vaccination. What the 1871-2 epidemic has done has been to make more certain that the claim originally made as to protection for the whole of life from one vaccination was an unsound claim, and that the protection afforded is one limited in its duration."

Q. 813. "But if the value of general vaccination were held to be demonstrated by the decline from this year, 1863, would not the large epidemic which followed immediately afterwards tend to falsify that conclusion?" "Not necessarily. You might, for example, during the present time put children into a hospital and claim that hospital isolation was the sole cause of diminishing scarlet fever; and then we might have an extremely virulent period of scarlet fever epidemic, when that would be disproved. But such epidemic prevalence of scarlet fever in spite of hospital isolation as one measure of prevention would be no ground for discontinuance of attempts to control the disease."

Q. 814. Referring to Q. 4,008, page 231, where Dr. Lyon Playfair asked: "I hold in my hand a circular from the Poor Law Commission Office, in Dublin, of the 20th of September, 1869. In that circular is the following passage: 'It is encouraging, however, to observe that the present total cessation of the disease in Ireland has been gradually approached since the compulsory vaccination of children has been put in force under the Act of 1863, the annual mortality by small-pox having been for many years previous about 1,000 deaths, on the average, representing, of course, a very much larger number of cases of attack and disfigurement. The course of decrease in the mortality has been as follows: In 1864 the number of deaths was 854; in 1865, 347; in 1866, 187; in 1867, 20; in 1868, 19; in the first quarter of 1869, 3, and in the second quarter none! Does that, in your opinion, represent fairly the action of the vaccination Act in Ireland?' To which Sir Dominic Corrigan replied: 'I am sure it does.' Would you agree with Sir Dominic Corrigan in that conclusion?" "I do not know that I could with reference to any passage picked out from the middle of the evidence of some man whom I have never met or seen, state whether I agreed with it or not; but I might say, as I have said before as regards England, that where we can study the details of the operation of the vaccination law, there is no question whatever in my mind that vaccination—recent vaccination above all things—is operative to prevent death from small-pox."

Q. 815. "I find that in 1871 in Ireland there were 665 deaths from small-pox; in 1872, 3,248; in 1873, 504; in 1874, 509, and in 1875, 565. Would the results of that epidemic in Ireland in 1871, 1872, 1873, 1874, and 1875 tend to alter in any way the opinion that the operation of the Act of 1863 was the cause of the decline in the preceding years?" "I would beg leave to answer that question at my further leisure. It contains a number of figures and hypotheses, and relates to a country of which I know nothing."

Q. 817. "At page 36 Dr. Seaton says: 'If I have not adverted to any influence which general sanitary conditions may have exercised on the small-pox mortality at home or abroad, it has been because the amount of any such influence is known to be wholly insignificant as compared with that of the presence or absence of effective vaccination in controlling small-pox mortality, especially in young children.' I take it that you would cordially agree with that?" "I would agree with that; and it is undoubtedly to the application of this great prophylactic measure to children (as contrasted with older persons) at a sufficiently early age that the remarkable saving of life exhibited by the facts brought together in this section is to be attributed."

[The reader is referred to the extract from Florence Nightingale's notes on nursing, given in THE HOMŒOPATHIC PHYSICIAN for March, page 78.]

Q. 818. "That being so, when you find so considerable a diversity in the figures as that shown by the table on page 19, of 13.6 per cent. under five years of age in the mainland rural districts, compared with 26.3 in the small towns, would you attribute that difference to the difference as regard extent and quality of vaccination, rather than to the difference as regards rural healthiness?" "I have already said that the fact of the aggregation of houses on areas, and of people in houses does affect small-pox to a certain extent, and it is well known that it is one of the reasons why small-pox is more frequent in towns than in rural districts."

Q. 819. "It is doubtless true that in the mainland rural districts the chances of introduction of small-pox are less, but why should that affect the proportion of deaths under five years of age to the total deaths?" "You are really asking me to give information as to circumstances upon which I have absolutely no information; if you will kindly limit yourself to the circumstances of England, on which I have information, I will willingly give it to you."

Qs. 775-6 (by the Chairman), are directed to elicit the fact that revaccinations by the public vaccinators were included in the returns made, and for which he is paid, but Dr. Thorne says: "He includes it in his register, but he makes no return of it to the medical department of the Local Government Board; it is, of course, included in his return to the guardians for the purpose of payment."

Q. 777. "But would it be included in his total of people vaccinated in a given time?" "*No, we never include it.* In fact, the figures I have before me and which relate to the very large increase of vaccination during the epidemic of 1870-71, distinctly do not include revaccination."

Q. 778. "Were not the adult revaccinations included in the figures before 1872, along with the primary infantile vaccinations, in the returns made to the Local Government Board?" * * * "*I am not aware that they were.*"

[The merest tyro in vaccinal statistics *knows* that they were so included.]

Referring (at Qs. 823 and 827, by Dr. Collins) to the answers given by him to Questions 777 and 778, he is asked, were the adult vaccinations "included along with the primary infantile vaccinations in the returns made to the Local Government Board; you apparently said that you were not aware that they were?" "I am sorry to say that matter does not come within my province at all, and I do not know."

Q. 828. "In answer to Q. 787, you said, referring to figures before 1851, 'I am absolutely confident that they are not procurable; not even the number of deaths from small-pox.' "

Q. 830. "Dr. Ogle directed our attention to a table which gave the deaths from 1847 to 1854 from small-pox at different ages?" "I would, of course, bow absolutely to Dr. Ogle; it comes within his province, and it does not come within mine. I may have been in error; indeed, I obviously have been in error, if Dr. Ogle gave you the information."

Q. 831. Calls his attention to Mr. Marson's table of the small-pox hospital, which shows that in every case of the vaccinated, those with one mark, two marks, three marks, and four marks, the mortality is higher in the period 1852 to 1867, than from 1836 to 1851.

Ans. "It is so."

Q. 832. "Could you give us any suggestion as to the explanation of that?" "I am sorry to say that I have *no knowledge* as to what was the quality of small-pox during those periods, or as to whether at one period more than another the severer cases only were admitted, and not the milder ones; in fact, I really have no knowledge that would explain it."

Q. 833. "But do you think that that points to the fact that small-pox was more virulent in the later than in the earlier period?" "I should say that it must have been more virulent among those patients that Mr. Marson had under treatment."

Q. 834. "Do you observe that in the *unvaccinated* the mortality is lower from 1852 to 1867, than it is from 1836 to 1851?" "I do notice it."

Q. 835. "Do you also notice that in the period 1852-67 the mortality of those stated to have been vaccinated, but having no cicatrix, is higher than in those confessedly unvaccinated?" "I do. Those two figures very often correspond; in fact, there are a great many people of whom it is affirmed that they have been vaccinated because they are afraid that something will happen to them for having broken the law."

Q. 838. "Do you think it at all possible that in that class the invisibility of the cicatrix and the virulence of the disease were in both cases referable to the abundance of the eruption?" "I must decline to speak as to periods, but I can answer you as regards present hospital experience. Patients now, *I am told,* hardly ever, if ever, come into a hospital with the eruption so abundant or at such a stage that it is not quite practicable to see whether there is a vaccination mark or not."

Q. 839. "Was Dr. Gregory, the predecessor of Mr. Marson at the small-pox hospital, an authority?" "*I do not know.*"

Q. 840. "You are aware that he was a predecessor to Mr. Marson?" "I am sorry to say that I had forgotten the fact."

Q. 841. Is a question founded on Dr. Gregory's statements as to the difficulty in determining who had been really vaccinated by *reason of the swollen and pock-covered condition of the arm at the time of the patient's admission* (*Medical and Chirurgical Trans.*, Vol. XXII.), and is here referred to for the purpose of enabling the following to be understood.

Q. 842. By Dr. Savory. "Mr. Marson's statements are explicit on the subject of the absence of the cicatrix?" "They are. The patients are stated to have been vaccinated, but having no cicatrix."

Q. 843. "There is no reference in Mr. Marson's writings to the difficulty of recognizing the cicatrix?" "I cannot recall that, but I am perfectly confident from what I knew of Mr. Marson, that he would never have put down 'having no cicatrix,' if he did not *know* that they had no cicatrix."

Q. 844. (By Dr. Collins) quoting a paper by Mr. Marson (*Medical and Chirurgical Trans.*, Vol. XXXVI, page 374): "Patients were never entered as vaccinated in the register unless the account of the vaccination was a tolerably clear one." "Then, again, do you think that the possibility of the doubt in the case of those stated to have been vaccinated, but, having no cicatrix, may have been due to the obscuration of the cicatrix by the abundance of the eruption?" "I should not like to base any answer on a mere quotation from a paper of that sort, without reading the paper."

With reference to Q. 845 (Jan. No., page 6), to the question, "How many marks Jenner required for complete protection?" Dr. Thorne's words in reply were: "I think I might mislead the Commission if I attempted to go into the early history of vaccination. I think I said on the last occasion that it is not a subject that I have studied in any detail."

Q. 846. "Are you aware that Jenner, in his *Further Observations*, page 109, said: 'A single cow-pox pustule is all that is necessary to render the variolus virus ineffectual?'" "I believe you are perfectly correct. I remember the statement, and it is one of a number of statements in which Jenner's experience was not, I assume, sufficiently lengthened and exhaustive to guide us at the present day."

Q. 848. (By Mr. Bradlaugh). "I understand Prof. Michael Foster to put it to you whether other authorities of later date on vaccination, had not repeated the declaration of Jenner, they having had opportunities of experience which Jenner had not?" "I am very sorry not to be able to give a definite answer, but the early history of vaccination is not a subject with which I profess to be well acquainted. My experience of vaccination mainly refers to comparatively recent years."

Q. 849. (By Dr. Collins). "I have here the last report of the Metropolitan Asylum's Board, which gives recent evidences for the year 1888, and I find on page 49, in the report from Dr. Birdwood, that he says: 'In revaccinating I have continued the practice of doing so in one place only. The results are quite as protective as if five or six vaccination scars had been produced.' No person employed at the ships has contracted small-pox during the year?" "I think that fully confirms what I have said, that even one good mark protects for a certain period. Nurses do not remain for the whole of their lives in these ships, and therefore the period of observation must necessarily have been a limited one."

Q. 850. In Vol. XXIV of the *Medical and Chirurgical Trans.*, page 33, Dr. Gregory says: "I think, from these cases, the cicatrix cannot be relied on as affording any certain test of the degree to which the constitution has imbibed an anti-variolous influence." "So that it would appear from Dr. Gregory, who apparently had as abundant opportunities of investigating as Mr. Marson, that his observations had led him to an opposite conclusion?" "I cannot pretend to go behind Dr. Gregory's facts: I never read his paper."

Q. 852. "Perhaps you are aware, with regard to the protection of nurses by revaccination, that the experiment was tried at the South Dublin Union Hospital of not revaccinating nurses?" "I believe that is doubted, but I do not happen to have the facts in my mind at present, and I cannot speak upon that point."

Q. 853. In *The Medical Press and Circular* for March 27th,

1872, in a paper read before the Surgical Society of Ireland, Mr. Frank Thorpe Porter, of the Small-pox Hospital, Dublin, says: "With reference to revaccination, I have no faith in it. Not one of the thirty-six attendants at the South Dublin Union Sheds has taken small-pox. Only seven of the number were revaccinated, and as the remaining twenty-nine enjoy the same immunity, wherein is the necessity of the operation?" Ans. "Does he happen to say whether they had had small-pox before?"

Q. 854. "He does not." "Then I should say that the evidence is of no value whatever without that information."

Here, to discredit the testimony of facts which do not favor his theory, Dr. Thorne assumes the autoprotection of small-pox, another theory, without any evidence to support it.

Q. 860. "Do you think that London is much better vaccinated, or much more vaccinated now than it was before the epidemic of 1871?" "I see, taking the returns for 1885, that seven per cent. of the births in the metropolis are unaccounted for as regards vaccination. That would, to some extent, answer one of your questions?"

Q. 861. "Could you tell whether the percentage unaccounted for would have been much larger before the epidemic of 1871?" "*I really do not know.* I know that a vast number of people in London were vaccinated and revaccinated during that epidemic, and, therefore, I should assume that there are now more vaccinated people in London than there were before the period of that epidemic."

Q. 862. "Can you give any estimate as to the percentage of Londoners who were vaccinated in 1871?" "*I cannot.*"

(Qs. 870 to 898 are noteworthy only as affording evidence of Dr. Thorne's skill in shuffling and evading unpleasant questions. I do not think it worth while to occupy Dr. James's space, or to weary the reader by reproducing them. If, after reading the preceding, any one still doubts Dr.

Thorne's ignorance of what he ought to know, or wishes to study the tricks of a shuffling witness, he can refer to the original blue-books.)

Q. 899 calls Dr. Thorne's attention to his answer to Q. 744, where he says: "So far as we have been able to gather, there is absolutely no other explanation for this marked reduction of small-pox mortality amongst children, except the mere fact that children are nearest in point of time to the date at which they were vaccinated?" He reasserts this opinion.

